# Estimating the annotation error rate of curated GO database sequence annotations

**DOI:** 10.1186/1471-2105-8-170

**Published:** 2007-05-22

**Authors:** Craig E Jones, Alfred L Brown, Ute Baumann

**Affiliations:** 1 School of Computer Science, University of Adelaide, South Australia, 5001; 2 Australian Centre for Plant Functional Genomics, Waite Campus, University of Adelaide, South Australia, 5064

## Abstract

**Background:**

Annotations that describe the function of sequences are enormously important to researchers during laboratory investigations and when making computational inferences. However, there has been little investigation into the data quality of sequence function annotations. Here we have developed a new method of estimating the error rate of curated sequence annotations, and applied this to the Gene Ontology (GO) sequence database (GOSeqLite). This method involved artificially adding errors to sequence annotations at known rates, and used regression to model the impact on the precision of annotations based on BLAST matched sequences.

**Results:**

We estimated the error rate of curated GO sequence annotations in the GOSeqLite database (March 2006) at between 28% and 30%. Annotations made without use of sequence similarity based methods (non-ISS) had an estimated error rate of between 13% and 18%. Annotations made with the use of sequence similarity methodology (ISS) had an estimated error rate of 49%.

**Conclusion:**

While the overall error rate is reasonably low, it would be prudent to treat all ISS annotations with caution. Electronic annotators that use ISS annotations as the basis of predictions are likely to have higher false prediction rates, and for this reason designers of these systems should consider avoiding ISS annotations where possible. Electronic annotators that use ISS annotations to make predictions should be viewed sceptically. We recommend that curators thoroughly review ISS annotations before accepting them as valid. Overall, users of curated sequence annotations from the GO database should feel assured that they are using a comparatively high quality source of information.

## Background

A major challenge facing bioinformatics today is how to effectively annotate an exponentially increasing body of publicly available sequence data. While using expert curators to assign functions to sequences might be considered to be the least error prone approach, this option is far slower than annotation by automated software approaches. On the other hand, automated function annotators often rely on curated sources of information from which to make predictions.

It is a commonly held view that curated sequence annotations are of better quality than automated annotations, however, the error rate of curated annotations can be significant. Estimates of the error rate of curated bacterial genome sequence protein and gene-name annotations lie between 6.8% and 8% [1,2]. The error rate of curated eukaryotic sequence annotations is far higher. Artamonova et al (2005) [3] examined the error rate of UniProt/SwissProt database annotations, consisting of five distinct types of annotation entries, and found an error rate of between 33% and 43%. As this database is widely considered to have a very high standard of curation, we might infer that other sequence databases have at least this annotation error rate, if not higher.

The quality of existing sequence annotations impacts on the quality of future sequence annotations through the commonly used practice of basing sequence annotations on sequence similarity. Errors in the use of sequence similarity based annotation strategies have been implicated in a number of commonly described annotation errors [4-6]. A common error is to put far too much emphasis on the importance of the best matching sequence, and not to review the significance of the match. Often this leads to false annotations due to a failure to recognise significant differences in protein domains [4,6] or open reading frames [5]. Overall, problems such as these lead to an increase in error rates of 5%–40% in annotations based on sequence similarity to previously annotated proteins [7].

There has been some discussion in the literature pin-pointing the importance of annotation error propagation [4,8]. Errors made by curators during the initial annotation of sequences can result in the generation of more errors in other data sources owing to the widespread use of sequence similarity-based annotation methods. For instance, misannotation of proteins to IMP dehydrogenase was found to propagate from the PIR-PSD database to SwissProt/TrEMBL, GenBank, and RefSeq [6]. The initial misannotation was caused by an error made while inferring the protein's name based on sequence similarity. In some cases where IMP dehydrogenase was erroneously assigned to sequences, the IMPDH domain was missing, whereas the CBS domains commonly found in IMP dehydrogenases were present [6]. Corrections to errors such as these may never occur. Meanwhile, new annotations may be based on erroneous annotations, and these in turn may have been based on erroneous annotations, and so on. Such 'chains of misannotation' [8] can lead to the progressive increase in annotation error rates.

Sequence annotation data generated by numerous projects has been submitted to the Gene Ontology (GO) Consortium and is available for download in various database releases [9]. A common use of this data source is to predict the function of novel proteins by using BLAST to find similar annotated sequences present in the database. This may be done by a biologist to find candidate GO terms for a sequence, or automatically by a growing body of electronic annotators [10-15]. The reliability of such inductive reasoning is determined by the correctness of the original sequence annotations. If the error rate of the source annotations is high then we would expect that annotation predictions based on them would be at least as high or higher. The current widespread use of sequence similarity based annotation methods simply assumes that input sequence annotations are correct. Without an understanding of the error rate of GO sequence database annotations it is not possible to assess the validity of making new sequence annotations based on evidence from existing sequence annotations.

In comparison to other forms of annotation, such as gene or protein name annotations, GO terms are used to describe the biological context of sequences. Indeed, GO term annotation has become the standard method by which functional information is attributed to sequence data. As far as the authors are aware, at the time of writing there is no published account systematically examining the error rate of curated GO term annotations. However, case-studies [4-7] and mathematical models [8] have shown that using sequence similarity to infer a new annotation is likely to be error prone. As each sequence annotation in the GO database has an evidence code, we can determine the impact of using sequence similarity based annotation on the error rate of curated sequence annotations directly.

As such the aims of this study are to a) develop an approach to estimating the error rate of GO term annotations, b) use this method to estimate the error rate of GO term sequence annotations submitted to the GOSeqLite database, c) and determine the impact, if any, of using sequence similarity based annotation methods on the error rate of annotations.

## Results

### Background

The GOSeqLite database (revision 3rd March 2006) was downloaded from the GO Consortium site [16], and imported into a MySQL database [17]. The database architecture is such that sequence annotation data are normalised, a single sequence record can have many associations (a GO term annotation), and each association has a related evidence record. This evidence record can be used to determine the basis on which the annotation was made. For instance, annotations that were based on sequence similarity to a previously annotated sequence are given the evidence code "ISS" (Inferred by Sequence Similarity).

If we were to select two sequences and their associated GO term annotations at random there are two broad reasons why their term annotations would differ. Firstly, two sequences may differ in their biological context, and differences in their GO term annotations reflect this. We will refer to this biologically relevant variation as 'semantic variation'. Secondly, annotations between two sequences may differ due to annotation errors. Such errors may be missing or incorrect GO term annotations. Owing to the fact that GO terms are related to each other via a directed acyclic graph (DAG), incorrect term annotations may be under-specialised (i.e. an ancestor) or over-specialised (a descendant) or be not directly related to the correct GO term that should have been present. This second form of variation we are referring to here as 'error variation'.

Instead of two sequences chosen at random, consider the case where we have a sequence, referred to as a 'query sequence', that is used in a sequence similarity search to find similar sequences against a reference set of sequences ('reference sequences'). Such a search might result in a large number of sequence matches between the query sequence and reference sequences. For each such sequence match it is possible to assign a precision to their matching annotations. If we consider that GO terms associated with the matching reference sequences are being used to predict the GO terms assigned to the query sequence, then the precision of the sequence match is:

P = n_m_/n_a_

where P is the precision of the sequence annotation, n_m _is the number of query and reference sequence GO terms that matched, and n_a _is the number of GO terms that the reference sequence is annotated with in the database.

This definition of precision allows only exact matches between query and reference sequence annotations to be considered correct. Because the GO is arranged in a DAG it could be possible to count reference sequence annotations as correct if they are within some number of edges of a query sequence term, as opposed to exact matching. However, previous work has demonstrated that increasing the permitted distance between the query and reference sequence terms dramatically amplifies the precision and recall, calling into question the applicability of these accuracy metrics under such conditions [14].

The precision of the annotations from any sequence match is determined by the semantic variation and error variation. For this reason, if the impact of the semantic variation can be controlled, the annotation error rate can be estimated by using a two-step method. Firstly, we must determine the relationship between annotation error rate and sequence-match annotation precision. To do this we will add annotation errors to reference sequence annotations at known rates, and use this to determine the relationship between precision and annotation error rate by means of linear regression. This will allow us to find a model that predicts the annotation error rate for a given precision value. Next, we must estimate the precision when no annotation error is present in the reference annotations. This precision value is referred to here as the 'maximal precision' because it is the highest precision that is possible for the sample of sequence matches. We assume that the semantic contribution to the precision is at its highest possible, and there is no error contribution, when the precision of the sample is equal to the maximal precision. Therefore, all differences between query and reference sequence annotations are due to differences in biological contexts. As any such estimate is likely to have a large impact on the final annotation error estimate, we will derive two independent approaches to estimating the maximal precision. Given that we know the basal precision of sequence-match annotations (the naturally occurring precision of sequence-match annotations) and the relationship between precision and annotation error rate, we can use the difference between the maximal and basal precisions to find the annotation error rate at the basal precision. This then is an estimate of the error rate of reference set annotations.

An important assumption here is that it is possible to compare the sample of sequence-matches used to derive the precision scores at artificially increased error rates, to the sample that is being used to generate a maximal precision estimate. At a BLAST expect value cut-off of 1e^-10 ^each query sequence has a potentially large number of matching reference sequences. For determination of precision scores we selected a sample of the total sequence-matches, where, for each query sequence in the query set, a single sequence-match was chosen that had the highest precision score. In other words, a single matching reference sequence was selected for each query sequence that had the high precision observed for all matches to that query sequence. It could be considered that this reference sequence is the best functional match to the query sequence. In this sample of sequence-matches, referred to as the 'highest precision sample', the semantic contribution to the precision must be at its greatest. It is probably a valid assumption that this sample also contains a representative error rate that can be used to estimate the annotation error rate of the entire population. The precision of the highest precision sample is determined by the highest observed semantic contribution and annotation error. As such, precision estimates from this sample are comparable to the maximal precision estimates. If no error is present in reference sequence annotations then the highest precision sample's precision scores would be approximately the same as the maximum precision estimate. Therefore, if we determine the relationship between precision and annotation error rate for the highest precision sample, the difference between the highest precision sample's precision and the maximal precision estimate can be used to derive an annotation error rate estimate.

### Maximal precision estimates

As described above, the maximal precision estimate is the maximum precision score possible for a sample of sequence-match annotations. When the precision is equal to the maximal precision estimate the semantic contribution to precision is maximised, and the annotation error is negligible. The maximal precision estimate will depend on the expect value cut-off of the BLAST search used. Here we adopted a cut-off of 1e^-10 ^for several reasons. Firstly, at this value both annotation error and semantic variation would be present. At lower expect value cut-off values, e.g. 1e^-100^, we would expect little semantic variation. Furthermore, annotation error would be difficult to detect as few query sequences would have significant matches except to themselves. Such self-matches, as determined by sequence id, are excluded from the analysis. Also, the derived error rate estimate could be considered more applicable to biologists when using this BLAST cut-off value, as it represents a fairly common use-case for biologists. However, it is important to note that most sequences found to be similar to a query sequence by BLAST at this cut-off value are not necessarily orthologous. Often they are simply protein sequences with one or more significant regions of similarity, such as a structural domain.

The importance of this estimate to the error estimation method cannot be overstated. The difference between precision at the naturally occurring error rate (basal precision) and the precision at zero error rate is used to directly estimate the annotation error rate via a function derived from regression coefficients. Therefore we have developed two independent approaches to estimating the maximal precision for a sample.

The first maximal precision estimation method is based on a number of simplifying assumptions concerning the distribution of semantic and error variation. If we assume that sequence-matches with no matching GO terms (i.e. a precision of 0) are purely due to semantic variation (i.e. significant differences in the biological contexts of sequences), we can then assume that cases that have a non-zero precision (i.e. at least one matching GO term between sequences) contain some semantic and some error variation. Therefore, if the error were to be removed from cases that have a non-zero precision, the precision could be 1. The assumption that all non-zero precision cases could have a precision of 1 if no error was present is very optimistic, and will result in a conservatively high maximal precision estimate. Using these assumptions we can derive a maximal precision estimate such that:

M_p1 _= N_m_/N

where M_p1 _is the first maximal precision estimate, N_m _is the number of sequence matches with at least one matching GO term, and N is the total number of sequence matches.

Note that the assumptions made above are considered to be fairly gross approximations of how error and semantic variation may be distributed. On the other hand it is unlikely that cases with a precision of 0 are due only to semantic differences between sequences. As the error rate increases more and more sequence matches are likely to have a precision of 0. For these reasons another maximal precision estimate was developed that is based on assumptions surrounding curated UniProt annotated sequences.

UniProt [18] is widely considered to provide 'gold standard' sequence annotations. The EBI-GOA [19] project produces electronically annotated and manually curated GO annotations. The manually curated annotations are accessible from the UniProt/Swiss-Prot database, and some are also available from the GO Consortium's website in releases such as the GoSeqLite database. The methods employed by GOA curators [20,21] results in annotations that we would expect to be of high quality, however it is unlikely that UniProt/Swiss-Prot sequence annotations contain absolutely no errors. However we can make this assumption for the purposes of obtaining an estimate of the maximal precision. As such the alternative estimate of the maximal precision, referred to as M_p2_, is simply the precision of cases where both sequences in the sequence match were annotated by UniProt/Swiss-Prot with self-matches, based on sequence id, excluded. In this case we are referring to UniProt/Swiss-Prot annotated sequences that are also present in the GoSeqLite database.

While both estimates of the maximal precision require significant simplifying assumptions concerning the relative weights and distributions of semantic and error variation, we might gain some reassurance in their reliability if they give similar results. We might expect that M_p1 _will generally provide a higher maximal precision estimate because it assumes that non-zero precision cases could have a precision of 1 where no error is present. Semantic variation will decrease the average precision of these cases, such that an average precision of 1 is unlikely. Alternatively M_p2 _will tend to provide a more generous estimate, as it presumes that UniProt/Swiss-Prot sequence annotations contain absolutely no annotation error. Existing evidence concerning the error rate of other forms of annotation [3] suggests that UniProt/Swiss-Prot GO term annotations show significant amounts of error. Any error in these annotations will tend to artificially lower the M_p2 _estimate. As such our two independent maximal precision estimates provide a useful range, and will in turn result in a range for annotation error estimates.

### Extrapolation to find annotation error rate at maximal precision

Once the relationship between precision and annotation error rate has been found using linear regression it is a simple matter to rearrange the standard regression prediction formula to find the annotation error rate corresponding to any precision:

X = (Y-B)/m

where X is the artificially added annotation error rate, Y is the maximal precision estimate, B is the regression constant, and m is the regression slope coefficient.

The above formula allows us to obtain the annotation error rate corresponding to a maximal precision estimate, given that we have already determined the values of regression coefficients. The annotation error rate (X) will be the error rate difference between the natural error rate (i.e. when the artificially added error rate is 0) and the annotation error rate corresponding to the maximal precision value. As a result the annotation rate estimate is:

where the annotation error rate estimate, E, is the absolute of value of X, where X is the artificially added annotation error rate.

### Analysis

#### Estimation of maximal precision scores

Maximal precision estimates were calculated for all experiments (table 1). Both maximal precision estimates were highly similar between cross-validation groups, and have very similar mean values (M_p1 _mean 0.882, sd 0.005; M_p2 _mean 0.851, sd 0.013). Both maximal precision estimates were derived independently using different methods. M_p1 _was calculated based on an assumption that non-zero precision cases could have a precision of 1 if no error was present while M_p2 _was based on UniProt to UniProt matches. However, the small difference between the means of these estimates might be taken as an indicator of accuracy. For the ISS annotation error estimation the maximal precision estimates were roughly half that of the non-ISS annotation error estimation experiment, however, both maximal precision estimates again had very similar values. Lower maximal precision estimates indicate that a much larger degree of semantic variation exists between the query and reference set annotations for this group.

**Table 1:** Maximal precision estimates, regression coefficients and annotation error rate estimates by experiment type.

**Experiment type**	**Maximal precision estimates**		**Regression coefficients**		**Annotation Error Estimates %**	
	**M**_**p1**_^**a**^	**M**_**p2**_^**b**^	**B**	**m**	**E**_**1**_^**c**^	**E**_**2**_^**d**^

Non-ISS annotation error						
Cross-validation group 1	0.882	0.841	0.760	-0.710	17	11
Cross-validation group 2	0.880	0.849	0.756	-0.703	18	13
Cross-validation group 3	0.885	0.845	0.767	-0.712	17	11
Cross-validation group 4	0.882	0.855	0.757	-0.704	18	14
Cross-validation group 5	0.886	0.836	0.757	-0.699	19	11
Cross-validation group 6	0.876	0.850	0.749	-0.698	18	15
Cross-validation group 7	0.891	0.867	0.768	-0.717	17	14
Cross-validation group 8	0.884	0.864	0.758	-0.709	18	15
Cross-validation group 9	0.885	0.873	0.760	-0.710	18	16
Cross-validation group 10	0.873	0.833	0.748	-0.697	18	12
ISS annotation error	0.442	0.443	0.305	-0.282	49	49

^a ^M_p1_, the maximal precision estimate derived from the ratio of non-zero precision cases to total in the highest precision sample.

^b ^M_p2_, the maximal precision estimate derived from the highest precision UniProt-UniProt sequence matches.

^c ^E_1_, the annotation error estimate based on M_p1_.

^d ^E_2_, the annotation error estimate based on M_p2_.

#### Calculation of regression coefficients

Data consisting of the precision of iterations at varying levels of artificially added GO term annotation errors were examined to determine a model for predicting precision from annotation error rate and vice versa. In all experiments, data showed a very high degree of linearity, with some gradual increase in variance as the annotation error rate increased. This increase was relatively small, and standardized scatterplots indicated that homoscedasticity (uniform variance in precision as error rate increased) was largely met. In all cases precision was normally distributed. Linear regression was used to determine the relationship between precision and annotation error for each experiment (table 1). In each the linear regression was statistically significant (p < 0.001, r^2 ^> 97.5). For the cross-validation groups there was very little variation in regression coefficient values (Constant (B) mean 0.758 sd 0.006; slope (m) mean -0.706 sd 0.006).

#### GO term annotation error estimates

Regression coefficients and maximal precision estimates were used to derive annotation error estimates for each sample (table 1). E_1 _and E_2 _are the error rate estimates derived from the maximal precision estimates M_p1 _and M_p2 _respectively. For non-ISS annotation error cross-validation groups, all have highly similar annotation error estimates (E_1 _mean 18%, sd 0.005; E_2 _mean 13%, sd 0.017). Both ISS annotation error estimates were found to be identical (E_1 _49%, E_2 _49%). Using the relative proportion of both types of annotation and their respective error rates, we estimate that the error rate of all curated GO term sequence annotations is 28% to 30%.

It should be noted that our research indicates that the expectation cut-off value of the BLAST match influences the error rate estimate (data not shown). Decreasing the expectation value cut-off resulted in an increase in the annotation error estimate. This appears to have occurred because, even though maximal precision and the regression constant coefficient (B) also decreased, there was a greater decrease in the regression slope coefficient (m), resulting in a greater annotation error rate at higher E-values. The E-value cut-off of 1e^-10 ^was chosen for this study as it reflects the upper value that would generally be used by biologists when attempting to find similar sequences. As such it provides a generous but realistic estimate of the error rate of GO term annotations.

## Discussion

The method developed and utilized here to estimate the GO term annotation error rate of GoSeqLite sequence annotations is based on a number of assumptions. Given that a query sequence has been found to be similar to a reference sequence (using BLAST) and both have an associated list of GO term annotations, we can calculate the precision of the sequence-match. This precision is determined by semantic variation (i.e. biologically relevant differences in the context of sequences) and error variation (errors made during the curation of sequences). We have used a number of assumptions to pry apart the effects of semantic and error variation to arrive at a method of estimating the error rate of GO term sequence annotations

The GO term annotation error rate estimates for the GoSeqLite database were found to be 13% to 18% for curated non-ISS annotations, 49% for ISS annotations, and 28% to 30% for all curated annotations. Other studies that examined different forms of sequence annotation (e.g. protein names) have found single forms of error that have accounted for between 6.8% and 8% of annotation errors alone [1,2]. Being only 2 to 3 times this value, a total error rate estimate of 13% to 18% for non-ISS annotations can be considered to be low. Furthermore, it appears that the error rate of curated GoSeqLite annotations as a whole compares favourably with a recently published estimated error rate of UniProt/Swiss-Prot sequence annotations of 33% to 43% [3]. As UniProt/Swiss-Prot annotations are considered to be of a gold-standard, and their estimated error rate is slightly higher than that estimated for GO database curated GO term annotations, we might draw the conclusion that GO database annotations also are of a very high standard of curation.

The magnification of sequence annotation errors through the use of ISS annotation methods has been identified as a possible major source of annotation error [4,8]. At first glance the GO sequence database could be used to examine this directly. Each ISS annotation has an associated evidence record, which in turn refers to a record in an external database. Sequence records also have a reference to an external database record. As such it is possible to indirectly find sequences that were related to records that were used as evidence for ISS annotations. Unfortunately there exists a many to many relationship between sequence and external database records, and as such it is not possible to identify specific sequence annotations as those used by curators as evidence for ISS annotations. For this reason we were unable to directly examine the effect of ISS 'error propagation' [8] on annotation error rates. However, the very fact that ISS annotations have a far higher error rate than non-ISS annotations indicates that ISS annotation strategies are inherently error prone. ISS annotation methods have been implicated in a number of cases of significant protein name annotation errors [1,2,5,7]. This study shows that ISS methods are also likely to dramatically increase the error rate of GO term annotations.

Before using GO term annotations all users should first be familiar with the meaning of GO evidence codes [9]. In particular it should be noted that there is a rough hierarchy of annotation quality. Curated, experimentally verified error codes (IDA, IEP, IGI, IPI, and IMP) are often considered the least error prone annotations. Alternatively, ISS, IEA, NR and ND evidence codes are considered the most error-prone. For this reason, the EBI GOA project avoids using GO annotations with these evidence codes for ISS-based annotation strategies [21]. This is likely to make ISS-based annotations far less error-prone. Other users might wish to consider placing similar constraints on their ISS-based annotation projects.

As far as the authors are aware, this is the first systematic study of GO term annotation error. We have found that the GO sequence database has a relatively low annotation error rate (28% to 30%), with non-ISS annotations having a much lower annotation error rate than ISS annotations (13% to 18% versus 49% respectively). As ISS annotations have approximately a 35% higher annotation error rate they should be viewed more suspiciously, and used more cautiously, than non-ISS annotations.

It is our recommendation that curators should only use ISS annotations after a thorough review. When a suitably similar sequence is found that is already annotated it would be prudent to examine the evidence concerning each annotation in detail to ensure that it is relevant in the current case. For instance, an annotated protein sequence may contain different protein domains to the sequence to be curated, and thus not all GO terms may be applicable. Because the error rate of ISS annotations is high, using sequence similarity to sequences as the basis for annotation, where that sequence was itself annotated based on sequence similarity, should be avoided. At the very least, the curator should search through these chains of annotations based on sequence similarity, to find the instances where annotations were made for other reasons, and determine whether that evidence is applicable to the current sequence.

There is a growing number of electronic annotators that predict GO terms to sequences based on sequence similarity to previously GO annotated sequences (e.g. [11,13,15]). It is common practice for these annotators to use ISS GO term annotations for the basis of predictions. The use of ISS annotations is likely to dramatically increase the rate of false predictions made by these annotators. For this reason, designers of electronic annotators should avoid the use of ISS annotations when developing prediction algorithms. Users of the output of electronic annotators that use ISS annotations to make GO term predictions should view the results sceptically.

## Conclusion

We have developed a method to undertake systematic analysis of GO term annotation error in sequence annotation databases, and used this to estimate the GO term annotation error rate of the GoSeqLite sequence annotation database. We found that the overall error rate is 28%–30%, and that GO term annotations not based on sequence similarity (non-ISS) have a far lower error rate than those that are, with error rates of 13%–18% and 49% respectively. Based on the available evidence, the overall error rate of the GoSeqLite database can be considered to be low. Due to the fact that the error rate of ISS annotations is relatively high, we recommend that curators using ISS annotated sequences as evidence for future annotations treat these with care to avoid propagating annotation errors. Furthermore, to ensure that the false prediction rate of electronic annotators is low, designers should avoid the use of ISS annotations when developing prediction algorithms. Indeed, it would be prudent to use curated, experimentally verified GO annotations as source data for annotators. We recommend against the unquestioning use of output from electronic annotators, especially those that use ISS annotated sequences to make GO term predictions.

## Methods

The aims of this study were to estimate the annotation error rate of curated GO term sequence annotations and to determine the impact on error rate of using sequence similarity based annotation approaches. To accomplish this, annotations were assigned to two groups based on their associated evidence code [9]. All annotations that had been assigned the evidence codes "Inferred by Curator" (IC), "Inferred from Direct Assay" (ID), "Inferred from Expression Pattern" (IEP), "Inferred from Genetic Interaction" (IGI), "Inferred from Mutant Phenotype" (IMP), "Inferred from Physical Interaction" (IPI), "Non-traceable Author Statement" (NAS), "No biological Data available" (ND), "Inferred from Reviewed Computational Analysis" (RCA), "Traceable Author Statement" (TAS), and "Not Recorded" (NR) were assigned to the "non-ISS annotations" group. All annotations that had been assigned the "Inferred by Sequence Similarity" (ISS) evidence code were assigned to the "ISS annotations" group

### Query and Reference Sequence Sets

In all cases we utilised a reference set of sequences and used BLAST to find similar sequences to a set of query sequences. All query sets were the complete sample of sequences that had non-ISS annotations associated (N = 59,251 sequences), and their annotations that were considered to be 'correct' for the purposes of precision scores, were their associated non-ISS annotations (N = 182,828 annotations).

In the case of estimating the annotation error rate of non-ISS annotations, 10-fold cross-validation was used. The entire query set was randomly broken into 10 equal sized groups. Each group was assigned to a query group, and the remaining 9 were assigned to a reference group. For example, the first cross validation group was assigned to query group 1, and was used as query sequences against a BLAST reference database consisting of cross-validation groups 2–10. The mean from all cross-validation groups was used as an estimate of the overall non-ISS annotation error rate estimate.

When estimating the annotation error rate of ISS annotations, the reference set is the set of sequences that only had ISS annotations (N = 54,551 sequences). This simplification allows the entire query set of non-ISS annotations and sequences to be used at one time. The reference set annotations are made up of ISS annotations only (N = 135,477 annotations). In this case we are comparing non-ISS sequence annotations against ISS sequence annotations.

### BLAST for Sequence Similarity

Formatdb was employed to create a BLAST custom database for each reference sequence set. The NCBI blastp application [22] was used to find all similar sequences above an expectation value threshold for each query and reference set. In all cases the expectation cut-off value was set to 1e-10. BLAST output was parsed and inserted into a MySQL database for further analysis. 1,536,168 and 1,685,408 matching reference sequences were found for the non-ISS and ISS annotation error rate estimation experiments respectively.

### Highest Precision Sample

Each query sequence had a potentially large number of matching reference sequences identified (mean = 26 for non-ISS error estimation, and 28 for ISS error estimation). SQL queries were written to extract the highest precision sample for each experiment. This involved assigning a precision to each query-reference sequence match according to the number of term annotations they had in common versus the number associated with the reference sequence. Then, for each query sequence the query-reference sequence match with the highest precision was selected for inclusion into the highest precision sample. This resulted in 59,251 sequence matches selected for the later error insertion experiment.

### Maximal Precision Estimates

The maximal precision estimates were determined for each group of BLAST results using SQL queries of the MySQL database. Select statements were written to find the number of sequence matches with at least one matching GO term (N_m_), and the total number of sequence matches for each group (N). The values were then used to calculate M_p1_. M_p2 _values were found with use of a select statement that found the average precision of GO term annotations where both the query and reference sequences were annotated by UniProt or UniProtKB. The M_p1 _estimate is based on the highest precision sample, while only matching sequences that were provided by UniProt are used to estimate M_p2_. These estimates were retained for later use with regression coefficients.

### Insertion of Annotation Errors

**Figure 1:**
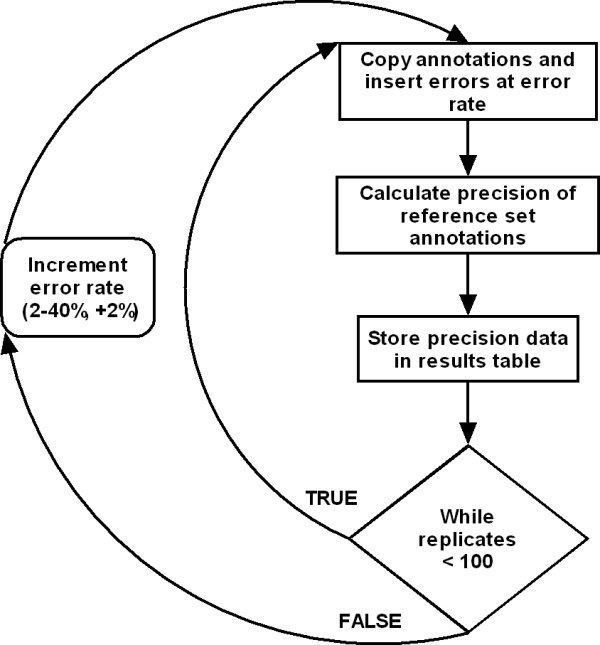
Insertion of annotation errors: Errors were randomly inserted into reference set annotations at a fixed error rate. The precision of reference set annotations for predicting the annotations of query sequences was determined, and the average precision at that error rate was recorded. This process was repeated 100 times for a given error rate value, after which the error rate was incremented. This process continued until data was obtained for artificially increased error rates of between 2% and 40%.

In order to determine the relationship between precision and annotation error rate for each highest precision sample, annotation errors were inserted into the reference set GO term annotations at known rates. Errors were added artificially to the annotations of matching sequences through an error-prone copy process. Firstly, a table with the same schema of the GoSeqLite annotation was created. The annotations belonging to high precision sample reference sequences were copied to this table, with a random chance of the annotation's GO term id being changed to an error flag during the copy operation. This random chance corresponded to the artificial error rate applied to the sample. Subsequently the precision of the highest precision sample sequence annotations at this error rate treatment was calculated. The error rate was examined between 2% and 40% inclusive, at 2% intervals. At each given error rate level 100 error prone annotation table copy replications were completed, and the precision of each replicate used in the final analysis. In total 20,000 replications were conducted over 20 treatment levels. A Java application was written to automate the process of error insertion (fig 1). Output was written to a MySQL database, which was later exported to a text file and imported into SPSS for statistical analysis.

This error insertion experiment was performed for both non-ISS and ISS annotation error estimation. In the case of non-ISS error estimation, the error insertion experiment was conducted independently for each cross-validation group. For ISS error estimation, the error insertion experiment was conducted once.

## Authors' contributions

CEJ undertook initial study design, software implementation, statistical analysis and interpretation, and drafted the initial manuscript. UB and ALB participated in the final study design, coordinated the study and contributed to the final manuscript.
